# Enhanced White Matter Fiber Tracts in Advanced Jazz Improvisers

**DOI:** 10.3390/brainsci11040506

**Published:** 2021-04-16

**Authors:** Kiran Dhakal, Martin Norgaard, Mukesh Dhamala

**Affiliations:** 1 Athinoula A. Martinos Center for Biomedical Imaging, Massachusetts General Hospital, Harvard Medical School, Boston, MA 02129, USA; kiran.neurophy@gmail.com; 2 Department of Physics and Astronomy, Georgia State University, Atlanta, GA 30303, USA; 3 School of Music, Georgia State University, Atlanta, GA 30303, USA; mnorgaard@gsu.edu; 4 Neuroscience Institute, Georgia State University, Atlanta, GA 30303, USA; 5 Georgia State-Georgia Tech Center for Advanced Brain Imaging, Georgia State University, Atlanta, GA 30303, USA; 6 Center for Behavioral Neuroscience, Georgia State University, Atlanta, GA 30303, USA; 7 Center for Nano-Optics, Georgia State University, Atlanta, GA 30303, USA; 8 Center for Diagnostics and Therapeutics, Georgia State University, Atlanta, GA 30303, USA

**Keywords:** music improvisation, creativity, connectivity, tractography, quantitative anisotropy, fiber integrity

## Abstract

Human cognition and behavior arise from neuronal interactions over brain structural networks. These neuronal interactions cause changes in structural networks over time. How a creative activity such as musical improvisation performance changes the brain structure is largely unknown. In this diffusion magnetic resonance imaging study, we examined the brain’s white matter fiber properties in previously identified functional networks and compared the findings between advanced jazz improvisers and non-musicians. We found that, for advanced improvisers compared with non-musicians, the normalized quantitative anisotropy (NQA) is elevated in the lateral prefrontal areas and supplementary motor area, and the underlying white matter fiber tracts connecting these areas. This enhancement of the diffusion anisotropy along the fiber pathway connecting the lateral prefrontal and supplementary motor is consistent with the functional networks during musical improvisation tasks performed by expert jazz improvisers. These findings together suggest that experts’ creative skill is associated with the task-relevant, long-timescale brain structural network changes, in support of related cognitive underpinnings.

## 1. Introduction

Examining the brain circuitry for creativity by using advanced brain imaging techniques has been an active field of research in recent years in efforts to understand the neural underpinnings of human creativity. Since jazz musical improvisation is one of the most complex forms of creative behavior, it has been used to understand real-time creativity, where revision is not possible. Recent neuroimaging studies on musical improvisation have identified brain regions and networks involved in musical improvisation and other creative domains such as literacy and drawing. As reviewed in contemporary literature [1,2,3], variations in brain activity and connectivity may be related to the heterogeneity of the participant’s background, their learned skills, experience, and creative expertise. In a recent neuroimaging study of advanced jazz musicians, we explored the divergent brain activation and connectivity patterns during musical improvisation and non-improvisation tasks. This functional magnetic resonance imaging (fMRI) study revealed higher regional activity in the inferior frontal gyrus (IFG), including the dorsolateral prefrontal cortex (dlPFC) and Broca’s area (BCA), lateral premotor cortex (lPMC), supplementary motor area (SMA), and cerebellum (Cb), with less functional connectivity in number and strength during musical improvisation compared with a pre-learned melody [4]. The directed functional connectivity further revealed that dominant information flow is from the lateral prefrontal cortex to the supplementary motor area in both conditions. The central roles of BCA, dlPFC, lPMC, and SMA have been widely discussed in creativity, both in domain-specific and domain-general abilities [3]. Although studies have reported consistent recruitment of the lateral prefrontal cortex and SMA in creative tasks, the network interaction patterns vary across studies. During musical improvisation, the real-time demands of the task most likely involve continuous generation with concurrent evaluation [5]. Acquired skills, training, experience, and knowledge, enable advanced improvisers to produce outstanding spontaneous performances while automatically controlling the interplay between perception, attention, and memory. This may result in an attenuated network interaction during improvisation, a state that has been referred to as hypofrontality [6]. These processes could result in the observed pattern of increased node activation and decreased connectivity during improvisation [4].

It is largely unknown how such divergent activity and connectivity patterns in experts emerge from the underlying brain structural organization and fiber architecture. While considering the dynamic functional states of activity and connectivity during creative performances, it is important to consider the behavioral consequences in the microscopic structural organization as well, particularly in white matter fiber properties. To characterize the brain structure, studies have investigated the variation in grey matter and white matter properties, associated with expert populations related to their cognitive skills, training/practice, experience, creativity, and behavioral expertise [7,8,9,10,11]. Despite the growing evidence of structural brain differences between musicians and non-musicians, whether and how the underlying white matter fiber properties reflect the neural activity and network interaction is not clearly understood. When comparing musicians with non-musicians, significant differences in diffusion properties have been reported in the corpus callosum, arcuate fasciculus, internal capsule, corticospinal tracts, superior longitudinal fasciculus, superior temporal gyrus, cerebellar peduncle, inferior-fronto-occipital fasciculus, uncinate fasciculus, inferior longitudinal fasciculus, and fiber tracts connecting posterior superior temporal gyrus and middle temporal gyrus [7].

Previous studies have mainly investigated structural pathways and direct fiber trajectories in terms of fractional anisotropy (FA), using diffusion tensor imaging (DTI), a method that relies on the movement of water molecules (i.e., how fast water molecules move along axonal fiber tracts) [12,13]. Variations in FA in white matter microstructure have been reported at both the individual and group levels [14,15]. However, inconsistency in findings across studies might be due to the types of musicians studied, including whether they are trainee or advanced level musicians; whether they are improvisers or non-improvisers, their skills, experience, and expertise; and the diffusion properties measured and the analysis methods used. The FA from the DTI method is an ensemble measurement and suffers from a partial volume effect, which may lead to inaccurate anisotropic measurement in complex fiber structures such as crossing fibers, free water diffusion in ventricles, and non-diffusive particles [16]. Here, we study the region-based and track-specific white matter fiber properties of advanced jazz improvisers and compared the findings with a non-musician control group. We examined the anisotropic diffusion properties in terms of quantitative anisotropy (QA), using the Q-Space diffeomorphic reconstruction (QSDR) approach [16,17] implemented in DSI studio toolbox (http://dsi-studio.labsolver.org/; accessed on 12 April 2020). The QA measure used in this study is different than the traditionally used fractional anisotropy (FA). The measurement of QA is based on the model-free nonparametric approach, which calculates the density distribution of water diffusion. QA is calculated from the peak orientations on a spin distribution function and is reported to have lower susceptibility to partial volume effects of crossing fibers and free-water, resulting in a better resolution with QA-aided tractography, which is known to outperform the FA-aided tractography [16,18]. Since QA is sensitive to the compactness of the fiber bundle, the normalization of QA (NQA) reduces the variability resulting in a stabilized spin-density measurement across subjects [17]. In addition to NQA measures, we examined the generalized fractional anisotropy (GFA), whose calculation is also based on the orientation distribution function like FA [19] and has a high correlation with FA [20]. 

The region-based and track-specific analysis is based on the functional network reported in our previous fMRI study of the same advanced jazz improvisers [4]. The region-based analysis includes the brain areas dlPFC in inferior frontal gyrus (IFG), lPMC in middle frontal gyrus (MFG), SMA, right cerebellum (RCb), and superior temporal gyrus (STG), whereas the track-specific connectivity analysis includes the fiber pathways connecting those brain areas. The region-based and track-specific fiber properties were investigated using the QSDR approach to test whether there were any significant differences in their diffusion anisotropies and how this structural property varied from the control group of non-musicians. We compared both the track-specific and region-based NQA measures of advanced jazz improvisers with the control group. Secondly, we explored whether the region-based and track-specific anisotropy measures of advanced jazz improvisers reflect the functional brain node and network activity patterns.

## 2. Materials and Methods

### 2.1. Participants

We studied two groups of healthy adults, matched as closely as possible in gender, age, and handedness. The “improvisation” group consisted of 20 advanced jazz improvisers (mean age ± standard deviation (sd) = 30.9 ± 13.3 years); the same participants were included in our previous functional magnetic resonance imaging study. For the improvisers, the criterion for participation was expertise in jazz improvisation [4]. Jazz improvisers had at least six years of professional experience (mean ± sd = 20.2 ± 12.8 years, see Table 1). Almost all the jazz improvisers had previous education in a university system school of music; average schooling years for all participants was 16.2 years (sd = 1.8 years). Improvisors were also required to know how to read music. Primary instruments were piano (*n =* 5), saxophone (*n =* 9), guitar *(n =* 1), trumpet (*n =* 2), drums (*n =* 1), trombone (*n =* 1), and bass (*n =* 1). The “control” group consisted of 20 non-musicians (mean age ± sd = 29.4 ± 4.4 years) who rarely played a musical instrument and had no previous music education.

All participants had normal, or corrected to normal, vision and reported normal neurological history. Participants provided written consent and were compensated for their participation. The Institutional Review Board for the joint Georgia State University and Georgia Institute of Technology Center for Advanced Brain Imaging, Atlanta, Georgia, approved the study. All research was performed in accordance with the relevant guidelines and regulations.

### 2.2. Behavioral Tests and MRI Scanning

Upon arrival at the testing site, participants provided informed consent and were familiarized with brain imaging procedures. All the participants completed practice sessions in a mock scanner to reduce anxiety and make sure they were comfortable with the MRI before going into the scanner for actual diffusion weighted imaging (DWI). Participants were instructed to remain still, not to move their heads or other parts of their body and focus on the central crossbar on the screen. During the data collections, instructions were displayed on a screen inside the scanner via the program “E-prime_V2.0.10.242” (https://www.pstnet.com/eprime.cfm; accessed on 19 March 2015). Details on the functional tasks can be found in the previous fMRI study [4].

### 2.3. DWI Data Acuisition and Image Processing

Diffusion-weighted imaging (DWI) data were acquired along 60 sampling directions. The b-value was 1000 s/mm^2.^ The slice thickness was 2 mm. A pair of images with no diffusion weighting (b0 images) was also acquired. We converted DWI data from DICOM to NIFTI format by using the dicom to nii (dcm2nii) toolbox part of the MRIcron. During this step, a *b*-value and *b*-vector file were generated along with the standard NIFTI file. Next, we performed standard eddy current correction using the FMRIB Software Library v6.0 processing software package (https://fsl.fmrib.ox.ac.uk/fsl/fslwiki/FDT/UserGuide; accessed on 12 April 2020) on DWI data for head motion and eddy correction. Next, we imported DWI data in DSI-Studio (http://dsi-studio.labsolver.org) and used a quality control procedure to ensure integrity and quality [21].

### 2.4. Data Analysis

#### Diffusion Weighted Imaging Data Analysis

For each participant, we estimated the anisotropic diffusion parameters mean GFA and mean NQA, using the Q-space diffeomorphic reconstruction (QSDR) approach [16,18] and deterministic fiber tractography [16,17,18] implemented in DSI Studio (http://dsi-studio.labsolver.org). These average diffusion indices are anisotropic measures. NQA is the normalized QA, a fiber-specific measure that quantifies for all fiber population, whereas the GFA is a generalized FA, a voxel-specific measure, shared by all the fiber population within a voxel [16,17,18]. QSDR is a model-free generalized Q-sampling imaging (GQI) approach, which calculates the density distribution of water diffusion at different orientations using a high-resolution standard brain atlas constructed from 90-diffusion spectrum imaging datasets in the ICBM-152 space. In QSDR, DSI Studio first calculates the quantitative anisotropy (QA) mapping in the native space and then normalizes it to the MNI QA map. QSDR also records the R-squared value between the subject QA and MNI QA map. We performed a Quality Check to make sure the “Neighboring DWI correlation,” the R-squared values between the subject QA and MNI QA map, were significant enough. The lowest “Neighboring DWI correlation” value was 0.8, which is significantly higher than the suggested R-squared value of 0.6.

A whole-brain tractography was performed. A deterministic fiber tracking algorithm [16,17,18] was used. The QA threshold was 0.12, which is higher than the suggested threshold value of 0.1. The angular threshold was randomly selected from 15 degrees to 90 degrees. The step size was chosen randomly from 0.5 voxels to 1.5 voxels. The fiber trajectories were smoothed by averaging the propagation direction with a percentage of the previous direction. The percentage was randomly selected from 0% to 95%. The parameters values were chosen randomly, which follows the parameter saturation approach avoiding false-negative results and is shown to have good test-retest reliability [22]. Tracks with a length shorter than 30 mm or longer than 200 mm were discarded. A total of 100,000 tracts were calculated. We applied Topology-Informed Pruning (TIP), which increases the accuracy by using the topology of a tractogram itself to identify the candidate of false connections for removal [23]. For each participant, GFA and NQA were estimated for all the possible tracts crossing five brain regions, namely the dlPFC, the lPMC, the STG, the RCb, and the SMA and the fiber pathways connecting these regions. The dlPFC in this study refers to a combined cluster of Brodmann area 9 (dlPFC) and 44 (Broca’s areas) in the left inferior frontal gyrus (IFG) identified in our fMRI study [4]. To ensure consistency across subjects, we normalized the QA measure by scaling the subject-wise maximum QA value to 1. Normalization of QA assumes that all the subjects have the same compactness of white matter. To avoid any bias among participants, an identical set of tracking parameters was used for jazz improvisers and control non-musicians. 

## 3. Results

### Track-Specific and Region-Based Fiber Tractography Results

**Figure 1:**
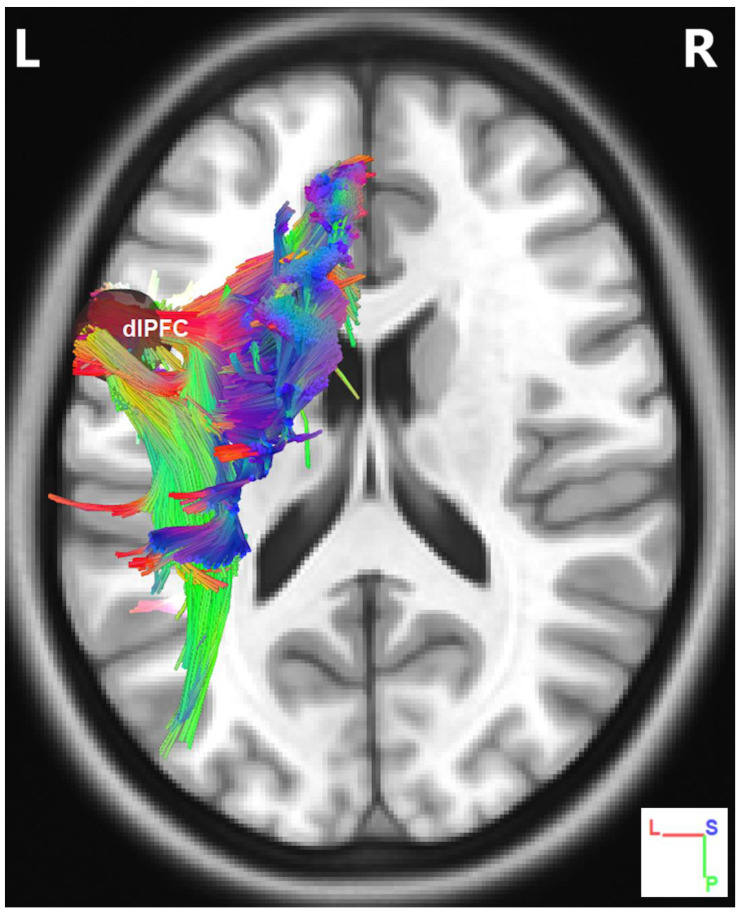
Fiber tracts crossing through seed region dlPFC for a representative participant. Here, fibers are colored-coded to represent their orientation, where “red” indicates fibers along the *X*-axis (i.e., left-right), “green” indicates fibers along the *Y*-axis (i.e., anterior-posterior), and “blue” indicates fibers along the *Z*-axis (i.e., inferior-superior).

**Figure 2:**
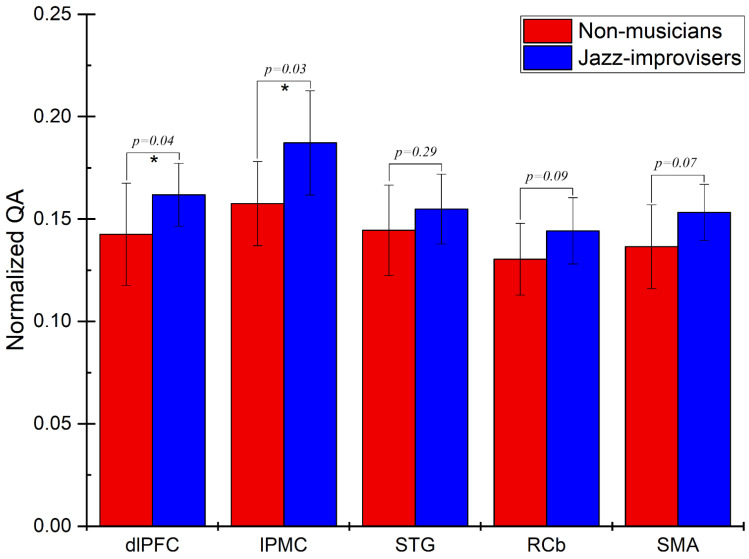
Region-based normalized quantitative anisotropy (NQA) for advanced jazz improvisers and non-musicians. Advanced jazz improvisers showed significantly higher NQA measures in frontal, and motor areas (dlPFC & lPMC), represented by a star * with the corresponding *p*-value

**Figure 3:**
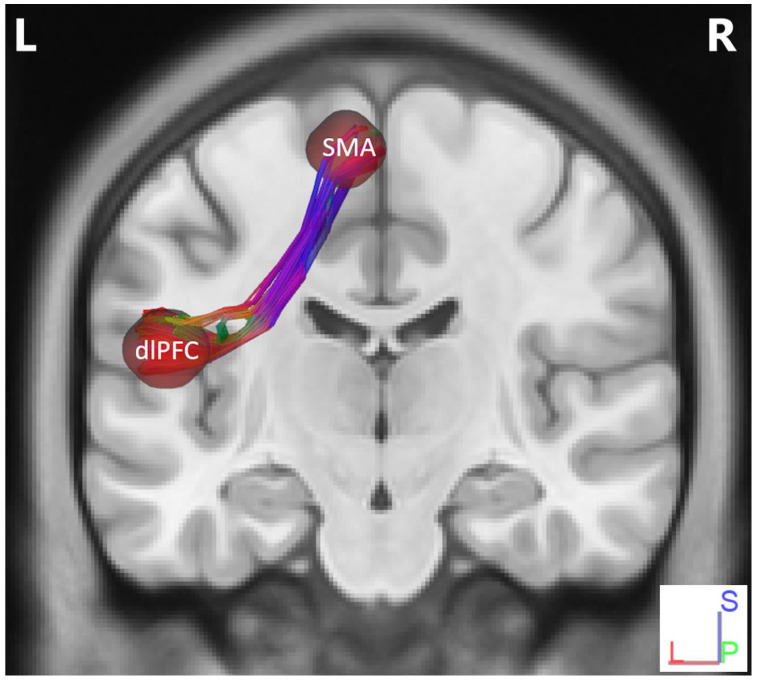
Fiber pathways between dlPFC and SMA for a representative participant. Here, fibers are colored-coded to represent their orientation, where “red” indicates fibers along the *X*-axis (i.e., left-right), “green” indicates fibers along the *Y*-axis (i.e., anterior-posterior), and “blue” indicates fibers along the *Z*-axis (i.e., inferior-superior).

**Figure 4:**
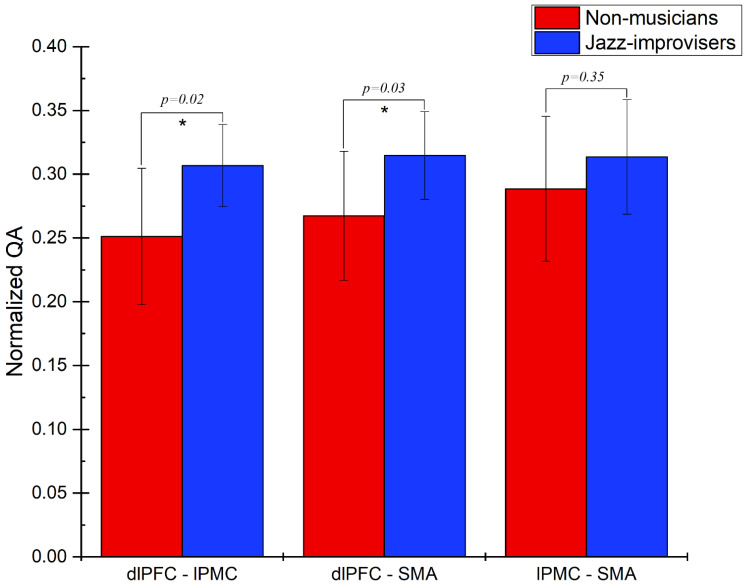
Track-specific normalized quantitative anisotropy (NQA) for advanced jazz improvisers and non-musicians. Advanced jazz improvisers showed significantly higher NQA measures in the prefrontal fiber pathways to the supplementary motor area, represented by a star * a with corresponding *p*-value.

**Figure 5:**
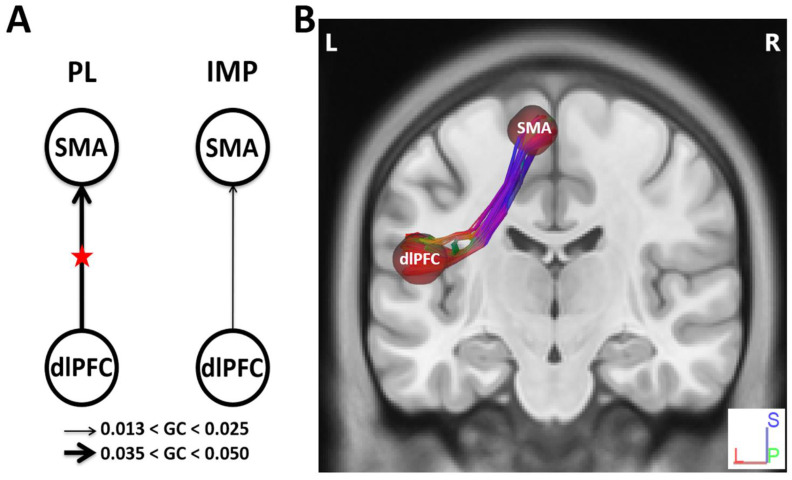
Schematic representation of functional and structural connectivity between the dorsolateral prefrontal cortex (dlPFC) and supplementary motor area (SMA). The functional network activity results are adapted from our previous fMRI study [4] of the same advanced jazz improvisers. (**A**) Granger causality from dlPFC to SMA during pre-learned and improvisation conditions. The red stars (left panel) represent an increase in network interaction directions (*p* < 0.05) when the causal strength during overall pre-learned is compared with overall improvisation. (**B**) Underlying white matter fiber pathway connecting dlPFC and SMA.

To determine diffusion parameters, we first performed whole-brain tractography, followed by limiting the white matter tracts to those passing through the 5 predefined regions of interest (ROIs)—namely the dlPFC, the lPMC, the STG, the RCb, and the SMA—and explored the fiber pathways connecting these regions. The selection ROIs was based on our previous fMRI study of the same advanced jazz improvisers, where these five regions showed higher brain activations during improvisation compared to pre-learned [4]. A detailed comparison of diffusion parameters GFA and NQA was performed on fibers crossing through the five specified regions and the underlying fiber pathways connecting them. A non-parametric statistical approach, the Wilcoxon signed-rank test, was used to test for significant differences.In Figure 1, we show fiber tracts crossing through region dlPFC for a representative participant, and in Figure 2, we show the region-based NQA for advanced jazz improvisers and control non-musicians. In Figure 3, we show the underlying fiber pathways between dlPFC and SMA for a representative participant, and in Figure 4, we show the track-specific NQA for advanced jazz improvisers and control non-musicians. In Figure 1 and Figure 3, fibers are colored-coded to represent their orientation, where “red” indicates fibers along the *X*-axis (i.e., left-right), “green” indicates fibers along the *Y*-axis (i.e., anterior-posterior), and “blue” indicates fibers along the *Z*-axis (i.e., inferior-superior).Advanced jazz improvisers showed significantly higher NQA measures in the lateral prefrontal and motor areas (dlPFC & lPMC) and the fiber pathways connecting dlPFC to motor areas (dlPFC-lPMC & dlPFC-SMA), whereas the GFA difference between advanced jazz improvisers and non-musicians was not significant. In Figures S1 and S2, in supplementary material, we also show regional and track-specific GFA for advanced jazz improvisers and non-musicians. Further, we checked the functional interaction pattern between dlPFC and SMA during jazz musical improvisation, as observed in our previous fMRI study [4], with the underlying fiber pathways connecting dlPFC and SMA. The dlPFC-SMA fiber pathway in advanced improvisers is enhanced with higher NQA measures compared to non-musicians. In Figure 5, we show the dlPFC-SMA fiber pathway together with the functional connectivity during jazz musical improvisation revealed by the Granger Causality (GC) analysis [24] in our previous fMRI study of the same advanced jazz improvisers [4]. The left panel (A) represents the information flow from dlPFC to SMA during pre-learned (PL) and improvised (IMP) conditions, whereas the right panel (B) represents the enhanced fiber pathway connecting dlPFC and SMA in advanced jazz improvisers. Interestingly, the connectivity is higher during the pre-learned condition compared to improvisation between these two areas.

## 4. Discussion

In this study, we investigated the track-specific and region-based fiber tractography of advanced jazz improvisers and compared the findings with a control group of non-musicians. We analyzed the anisotropic diffusion properties GFA and NQA for fibers crossing previously defined brain regions and the underlying fiber pathways connecting them. We found the region-based fiber crossings and the underlying white matter pathways in advanced improvisers characterized by higher fiber integrity (NQA), especially in the frontal motor regions and the connecting fiber pathways as compared to non-musicians. When we checked the pattern of the functional interaction between dlPFC and SMA during jazz musical improvisation, as explored in our previous study [4], with the underlying fiber pathways connecting dlPFC and SMA, we found the dlPFC-SMA fiber pathway in advanced improvisers is enhanced with higher NQA measures compared to non-musicians. These results suggest the white matter fiber properties have behavioral consequences that reflect the functional architecture of creative expertise. On the other hand, we found no significant differences in GFA measures in the frontal motor regions and the connecting fiber pathways as compared to non-musicians.

Previous DTI studies of musicians have mainly discussed the diffusion properties of the underlying white matter microstructure in terms of FA using the probabilistic tractography methods. Most of the studies used the long-range white matter tracts or literature driven brain regions as their regions of interest. However, these studies have yielded somewhat inconsistent findings, as some report high FA values [25,26,27] and others report low FA values [28,29] in musicians and other creative individuals, (for a review, see Moore 2014). Such inconsistency may be due to several factors including, methods, types of musicians, experience, expertise level, training, skills, and creative potential. In this study, we examined the NQA instead of FA using the deterministic fiber tractography, the Q-Space diffeomorphic reconstruction (QSDR) approach [16,17], which calculates the density distribution of water diffusion at different orientations. We also examined GFA using the same deterministic QSDR method, which is similar to FA. However, the NQA measure used in this study is different than the traditionally used fractional anisotropy (FA). QA is reported to have lower susceptibility to partial volume effects of crossing fibers and free-water, resulting in a better resolution with QA-aided tractography, which outperforms the FA-aided tractography [16,18]. Since QA is sensitive to the compactness of the fiber bundle, the normalization of QA (NQA) reduces the variability resulting in stabilizing the spin-density measurement across subjects [17,22]. On the other hand, generalized FA (GFA) suffers from the same partial volume effect as FA, and value decreases in fiber crossing or voxels with partial volume effect [17,18].

We found significantly higher NQA in several brain regions of advanced improvisers compared to non-musicians, specifically in dlPFC within the IFG, and lPMC in MFG, both of which were associated with higher regional activity during musical improvisation as we reported in our fMRI study of the same advanced jazz improvisers [4]. In addition, the fiber pathways connecting these regions are characterized by higher NQA, specifically the fiber pathways between the frontal and motor regions. NQA in the fiber pathway between dlPFC and SMA, the connection associated with the information flow during musical improvisation, is significantly higher in advanced improvisers compared to non-musicians, suggesting that the underlying fiber integrity may serve as the basis for functional interaction. On the other hand, we found no significant differences in regional GFA measures and the connecting fiber pathways in jazz improvisers as compared to non-musicians in the frontal motor regions (dlPFC, lPMC & SMA) and fiber pathways connecting these regions. This could be due to the limitations of generalized FA (GFA) that suffers from the partial volume effect and value decreases in fiber crossing or voxels with partial volume effect [17,18].

Neurocognitive processes underpinning musical improvisation include fitting responses to an overall architectural structure, first selecting individual auditory and motor chunks, and then combining these chunks into an action chain [30,31]. The areas that exhibited increased activation during improvisation in our previous study were dlPFC, lPMC, SMA, and RCb [4]. The dlPFC is associated with goal-directed behaviors that are consciously monitored, evaluated, and corrected and is a central part of the executive control network (ECN). Specifically, dlPFC is involved in inhibiting habitual responses [32]. The involvement of left dlPFC during musical tasks presumably indicates top-down control, attentional monitoring, and evaluation [33,34]. The activation of the motor planning areas lPMC and SMA during improvisation may be due to the process of selecting single motor acts or single sensorimotor associations associated with the hierarchical organization of the human behaviors [35].

Concerning the connectivity between ECN and motor regions, the elevated white matter fiber anisotropy of these regional crossings in advanced jazz improvisers may underline the increased performance and activity during creative cognition, working memory tasks, practice, and training [26,36,37]. Further, the enhanced frontal-motor fiber pathways characterized by higher NQA may be due to these behaviors. In our previous fMRI study of the same advanced jazz improvisers, there was less causal effect during improvisation compared to pre-learned conditions, but the net information flow was always from dlPFC to SMA in both conditions [4]. The structural architecture of the advanced jazz improvisers may subserve as the basis for their functional interaction during musical performances. With the highly enhanced underlying white matter fiber pathways, the output of the executive network evaluation may need minimal communication to motor regions during real-time musical improvisation compared to prelearned performance. In other words, enhanced fiber tracts in experts may subserve as the basis of efficient execution of their overlearned skills and strategies when it comes to creating seemingly novel feats.

Finally, we also discuss here several potential limitations in this study. First, NQA measures yielded significant findings compared to the GFA measures, but the accuracy of the measures could not be directly estimated. However, NQA-aided tractography, which is known to filter out noisy fiber tracts and yield results in a higher spatial resolution due to its lower susceptibility to partial volume effects [18], is a better approach for examining fiber properties. Another limitation is that in our sample of advanced experts, the experts’ performances were not variable and could not be correlated with NQA measures. Future studies with larger samples of experts with different years of improvisation practice can potentially capture this variability and quantify the relationship with improvisational behavioral measures. The goal here was to compare the differences between expert jazz improvisers and non-musicians (controls), and explore the underlying white matter architecture consistent with the functional interaction pattern as observed in improvisatory task execution. To further examine the structural basis of musical creativity, future studies would benefit from comparing the samples of advanced improvisers from different creative domains and explore the whole brain functional and structural architecture. Brain connectivity specifically related to improvisation could potentially be identified by comparing improvising musicians to non-improvising musicians. Further, expanding the investigations in other creative domains like literary creativity, drawing creativity, dance etc. might extend the understanding of the structural organization in domain-specific and general creativity.

## 5. Conclusions

In this study, we investigated the white matter fiber properties of advanced jazz improvisers by conducting a QSDR deterministic tractography analysis. The elevated NQA measures in advanced jazz improvisers indicate enhanced task-supportive structural connectivity in improvisers compared to non-musicians. The enhanced fiber pathways connecting frontal and motor regions in advanced jazz improvisers could explain related brain activity and connectivity patterns during improvisatory task execution, which altogether points to the neural basis of experts’ real-time creative performance.

## Figures and Tables

**Table 1:** Age, the primary musical instrument, and years of experience (jazz experience) of the advanced jazz improvisers in this study.

Participant No.	Age (Years)	Years of Experience (Jazz Improvisation)	Primary Instrument
01	31	24	Piano
02	57	50	Piano
03	41	31	Saxophone
04	43	34	Piano
05	33	22	Piano
06	20	6	Guitar
07	22	10	Saxophone
08	26	15	Saxophone
09	41	33	Saxophone
10	23	11	Saxophone
11	19	10	Saxophone
12	26	18	Piano
13	30	18	Contra/Double Bass
14	21	12	Trombone
15	28	14	Drum Set
16	23	14	Saxophone
17	23	12	Saxophone
18	42	33	Trumpet
19	38	28	Saxophone
20	22	11	Trumpet

## Data Availability

The data presented in this study are available on request from the corresponding author.

## Supplementary Materials

The following are available online at https://www.mdpi.com/article/10.3390/brainsci11040506/s1, Figure S1: Region-based subject-averaged generalized fractional anisotropy (GFA) for advanced jazz improvisers and control non-musicians. No significant differences observed in regional GFA measures except in fiber crossing of STG; Figure S2: Tracks-specific subject-averaged generalized fractional anisotropy (GFA) for advanced jazz improvisers and non-musicians. No significant differences observed in GFA measures in the prefrontal fiber pathways to the supplementary motor area.
