# Supplementary material for: Enhanced White Matter Fiber Tracts in Advanced Jazz Improvisers
Source: Brain Sci. 2021 Apr 16;11(4):506. doi: 10.3390/brainsci11040506 (PMC8073640; doi:10.3390/brainsci11040506)
Supplement: Supplementary file 1 [file brainsci-11-00506-s001.zip › brainsci-1177262-supplementary.pdf]

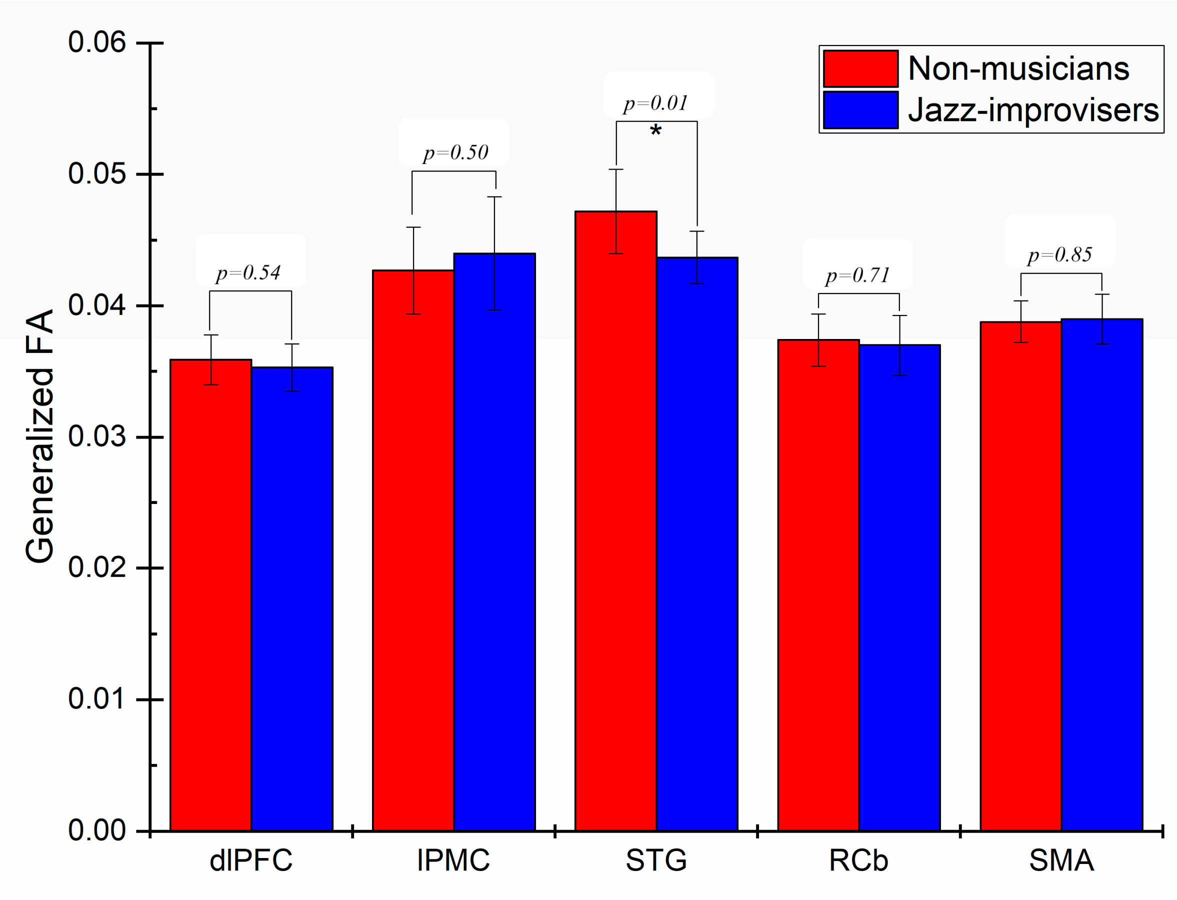

Figure S1. Region-based subject-averaged generalized fractional anisotropy (GFA) for advanced jazz improvisers and control non-musicians. No significant differences observed in regional GFA measures except in fiber crossing of STG.

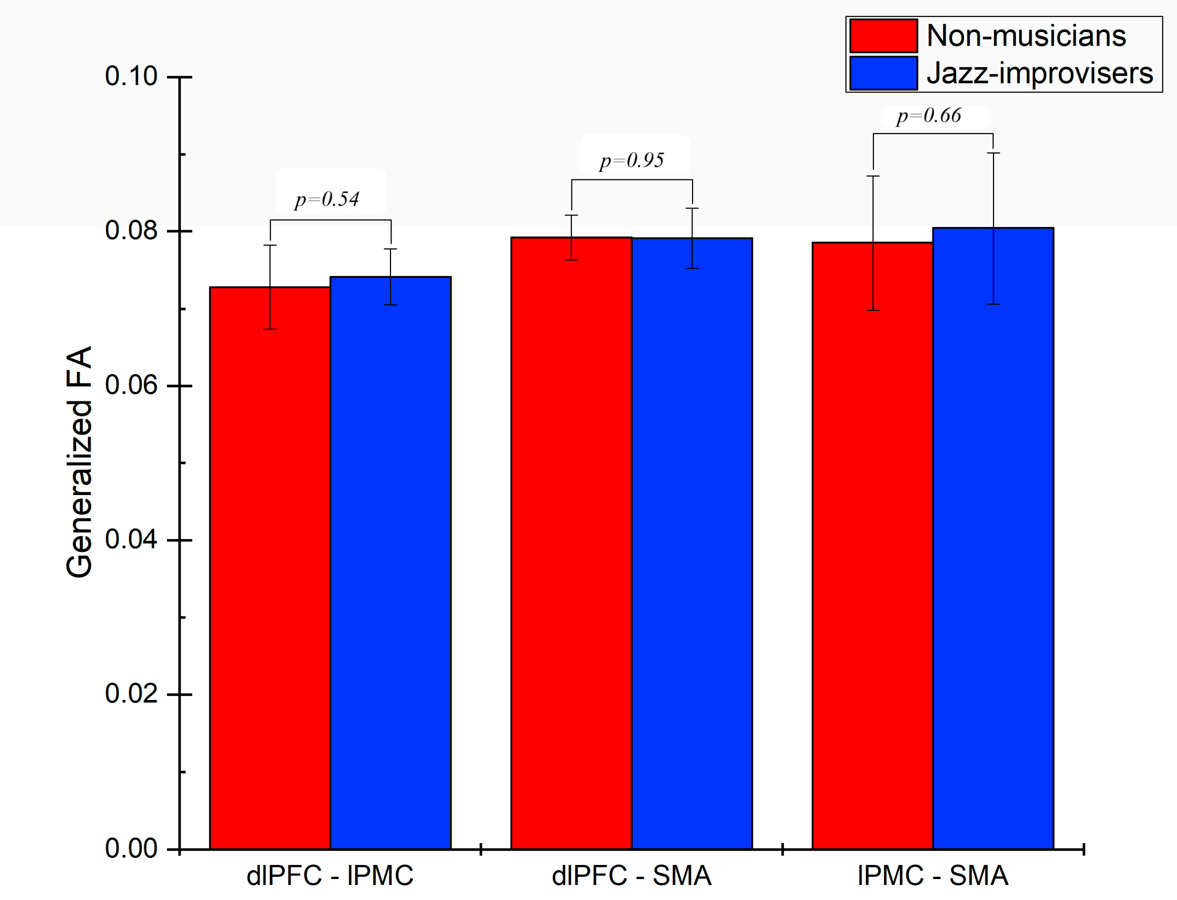

Figure S2. Tracks-specific subject-averaged generalized fractional anisotropy (GFA) for advanced jazz improvisers and non-musicians. No significant differences observed in GFA measures in the prefrontal fiber pathways to the supplementary motor area.
